# Vildagliptin Attenuates Hepatic Ischemia/Reperfusion Injury via the TLR4/NF-*κ*B Signaling Pathway

**DOI:** 10.1155/2018/3509091

**Published:** 2018-10-14

**Authors:** Iman O. Sherif, Nora H. Al-Shaalan

**Affiliations:** ^1^Emergency Hospital, Faculty of Medicine, Mansoura University, Mansoura 35516, Egypt; ^2^Chemistry Department, College of Science, Princess Nourah bint Abdulrahman University, Riyadh 11671, Saudi Arabia

## Abstract

The Toll-like receptor-4 (TLR4)/nuclear factor kappa B (NF-*κ*B) signaling pathway is vital in the pathogenesis of hepatic ischemia/reperfusion (HIR) injury. Dipeptidyl peptidase-4 (DPP4) inhibitors exert protective effects on IR injury of the kidney, heart, and lung; however, their effect on the liver is still unknown. Thus, the purpose of this study was to examine whether pretreatment with vildagliptin (Vilda), a DPP4 inhibitor, produces hepatic protection against IR injury and to investigate its influence on TLR4/NF-*κ*B signaling in a rat model. Thirty male Wistar rats were divided into 3 groups: the sham group: subjected to a sham operation and received normal saline; the HIR group: subjected to HIR and received normal saline; and the Vilda + HIR group: subjected to HIR with pretreatment of 10 mg/kg/day Vilda for 10 days intraperitoneally. Hepatic ischemia lasted for 45 minutes followed by 3-hour reperfusion; then blood and liver samples were collected for biochemical and histopathological examination. The HIR group produced a significant increase in serum alanine aminotransferase (ALT), aspartate aminotransferase (AST), hepatic malondialdehyde (MDA), nitric oxide (NO), and tumor necrosis factor alpha (TNF-*α*) levels and a significant reduction in the hepatic catalase level in comparison to the sham group. Moreover, a significant upregulation of gene and protein expressions of TLR4, NF-*κ*B, and high-mobility group box-1 (HMGB1) along with caspase-3 protein expression was observed in the HIR group when compared with the sham group. Histopathological examination of the liver from the HIR group showed necrosis, sinusoidal congestion, hemorrhage, and hepatocyte degeneration. Administration of Vilda ameliorated the biochemical and histopathological changes caused by HIR. Vildagliptin showed for the first time a hepatoprotective effect in HIR injury through downregulation of TLR4/NF-*κ*B/HMGB1 and caspase-3 hepatic expressions.

## 1. Introduction

Hepatic ischemia/reperfusion (HIR) is commonly performed in clinical liver surgeries including liver transplantation [1]. The pathogenesis of HIR injury is a complex process involving not only necrosis and apoptosis but also oxidative stress, inflammation, and dysfunction of the liver cells [2, 3].

The Toll-like receptors (TLRs) constitute a family of transmembrane receptors that have an important role in the detection of the microbial infection through innate immune system activation [4]. The most studied member in this family is TLR4 which can detect the presence of endogenous molecules secreted from damaged or ischemic tissues [5, 6]. TLR4 signaling was reported to be activated by high-mobility group box-1 (HMGB1), heparan sulfate, and others [7–9].

Moreover, TLR4 has been recognized as a mediator of inflammation and organ injury in different models including hepatic [8, 10], renal [11], and pulmonary [12] I/R injury. Furthermore, the TLR4/NF-*κ*B signaling was found to play a significant role in the pathogenesis of HIR injury [13].

Dipeptidyl peptidase-4 (DPP-4) is a serine exopeptidase enzyme responsible for degradation of incretins as glucagon-like peptide-1 (GLP-1) and gastric inhibitory polypeptide (GIP) [14]. GLP-1 reduces the blood glucose by stimulation of insulin secretion; thus, the inhibitors of DPP-4 that reduced the GLP-1 cleavage are used for type 2 diabetes treatment [15, 16]. DPP-4 inhibitors are a class of diabetic drugs that include sitagliptin, saxagliptin, vildagliptin (Vilda), and linagliptin [17].

It was found that DPP-4 inhibitors have the ability to protect the kidney, heart, and lungs against I/R injury [18]. The use of Vilda improved the renal function and ameliorated the renal tubular necrosis and apoptosis in a rat model of renal I/R injury via its antiapoptotic and antioxidant activities [19]. Recently, the cardioprotection of Vilda was determined in diabetic hearts exposed to I/R injury by attenuating the myocardial I/R damage through reduction of oxidative stress and mitochondrial dysfunction [20]. Moreover, the neuroprotective effect of Vilda was evident against cerebral ischemia through reduction of the apoptotic executive caspase, caspase-3 protein expression, and modulating oxidative stress markers in the brain [21].

However, there are still no findings on the ability of DPP-4 inhibitors including Vilda to protect the liver against I/R injury. Thus, this study was designed to investigate whether Vilda treatment could protect the liver against I/R injury and to explore its effect on the TLR4/NF-*κ*B signaling pathway and the consequent inflammatory and apoptotic responses that are implicated in the development and progression of the HIR injury.

## 2. Materials and Methods

Male Wistar Rats weighing 250–300 g were used in this study and were kept under controlled conditions with a 12-hour light/dark cycle and a free access to standard chow and tap water ad libitum. Animals were handled following the guide for the Care and Use of Laboratory Animals as adopted by the International Accreditation Organization and approval from the Animal Ethics Committee of Nile Center for Experimental Researches, Egypt, with approval no. 2018-008.

### 2.1. Experimental Design

The animals were divided into 3 groups with each group having 10 animals—group 1 (sham group): subjected to a sham operation and received normal saline; group 2 (HIR group): subjected to HIR and received normal saline; and group 3 (Vilda + HIR group): subjected to HIR with pretreatment of intraperitoneal injection (ip) of 10 mg/kg/day Vilda (Galvus, Novartis Pharma Stein AG., Switzerland) for 10 days and 15 min prior to ischemia in the day of the operation [19].

### 2.2. Hepatic Ischemia/Reperfusion Injury Operation

**Figure 1:**
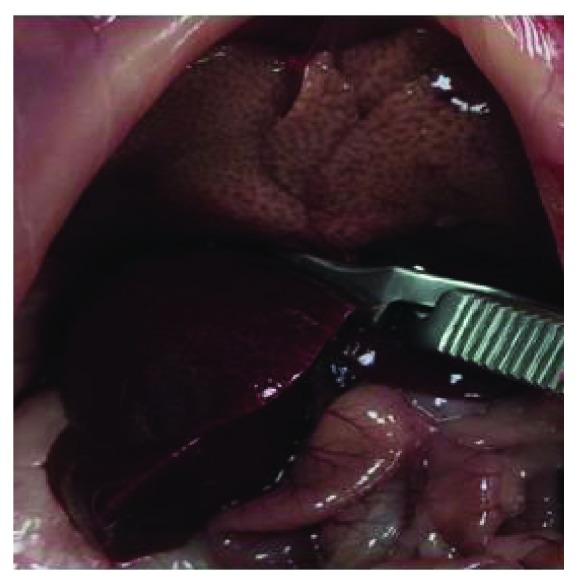
Rat liver after clamping of the portal triad with hepatic ischemia in the median and left hepatic lobes.

The HIR injury was performed as described in previous studies [22, 23]. Rats were anesthetized with a mixture of ip 80 mg/kg ketamine (Ketamax-50, Troikaa Pharmaceuticals Ltd., Gujarat, India) and 10 mg/kg xylazine (Xyla-Ject®, Adwia Co., 10th of Ramadan, Egypt) [24, 25]. A midline abdominal incision was made after shaving the rats' abdominal front wall and disinfected by the povidone-iodine solution. The portal triad including the hepatic artery, portal vein, and bile duct was occluded by using a Bulldog clip to block the blood supply to the median and left liver lobes producing 70% of hepatic ischemia which was manifested by the color change in the affected lobes as presented in Figure 1. The period of hepatic ischemia lasted for 45 minutes while the period of reperfusion lasted for 3 hours. The sham group underwent the same procedure but without making vascular occlusion.

At the end of the reperfusion period, the clip was removed and blood flow was restored and reperfusion was confirmed by changing the liver color. Rats were sacrificed under anesthesia by exsanguination through blood and tissue collection.

### 2.3. Blood Sampling

Blood was collected and centrifuged at 3000 rpm for 5 minutes. Serum samples were separated and stored at −20°C for the determination of the liver function.

### 2.4. Harvesting of Liver Specimen

Parts of ischemic liver tissue were immediately removed and immersed in liquid nitrogen and kept at −80°C for oxidative stress marker determination in addition to enzyme-linked immunosorbent assay (ELISA), real-time quantitative polymerase chain reactions (qPCR), and Western blot techniques. Other parts of liver lobes were kept in 10% phosphate buffered formalin for histopathological examination.

### 2.5. Hepatic Tissue Homogenate Preparation

A part of the liver was ice cooled, homogenized in 10-fold phosphate buffer (pH 7.4), and then centrifuged at 600 × g for 10 minutes at 4°C. The supernatant, referred to as homogenate, was stored at −80°C until measurement.

### 2.6. Liver Function Tests

To assess hepatic function, serum alanine aminotransferase (ALT) and aspartate aminotransferase (AST) were determined by a colorimetric method using commercially available kits provided by Bio-Diagnostic Company, Giza, Egypt.

### 2.7. Hepatic Oxidative and Nitrosative Stress Markers Determination

Hepatic malondialdehyde (MDA) activity was determined using MDA assay kit (Sigma-Aldrich, St. Louis, MO, USA) based on the reaction of MDA with thiobarbituric acid to form a colorimetric product which is proportional to the content of MDA and measured at 532 nm [26]. Moreover, the level of catalase was measured in hepatic tissue homogenate using catalase assay kit (Cayman Chemical, Ann Arbor, MI, USA) in which the formaldehyde produced was measured colorimetrically with purpald at 540 nm [27] while the hepatic nitric oxide (NO) was assessed using NO assay kit (BioVision, Milpitas, CA, USA) through colorimetric method by measuring total nitrite/nitrate content using Griess reagent [28].

### 2.8. Hepatic Inflammatory Marker Estimation

The inflammatory marker as tumor necrosis factor alpha (TNF-*α*) in hepatic tissue homogenate was determined using rat TNF-*α* ELISA kit (Cloud-Clone Corp., Houston, TX, USA) according to the manufacturers' protocol.

### 2.9. Real-Time Quantitative PCR (qPCR) for Gene Expressions of TLR4, HMGB1, and NF-*κ*B

From the liver tissues of the three groups, total RNA was isolated with RNeasy Mini Kit (QIAGEN, Valencia, CA, USA) and then analyzed for quantity and quality (A260/A280) with Beckman dual spectrophotometer. For quantitative expressions of TLR4, HMGB1, and NF-*κ*B, 10 ng of the total RNA from each sample of liver tissue was used for reverse transcription to cDNA synthesis by using High-Capacity cDNA Reverse Transcriptase kit (Applied Biosystems, Foster City, CA, USA). After that, the cDNA was amplified with the SYBR Green PCR Master Mix Kit (Applied Biosystems) using the StepOne instrument as follows for the amplification step: 10 minutes at 95°C for enzyme activation followed by 40 cycles of 15 seconds at 95°C, 20 seconds at 60°C, and 30 seconds at 72°C. Changes in the expressions of the examined genes were normalized relative to the mean critical threshold (CT) values of the housekeeping gene glyceraldehyde 3-phosphate dehydrogenase (GAPDH) via the ΔΔCt method. The primer sequences used were as follows: TLR4 (forward primer: 5′-CGGAAAGTTATTGTGGTGGTGT-3′, reverse primer: 5′-GGACAATGAAGATGATGCCAGA-3′), HMGB1 (forward primer: 5′-CCGGATGCTTCTGTCAACTT-3′ reverse primer: 5′-TTGATTTTTGGGCGGTACTC-3′), NF-*κ*B (forward primer: 5′-AGAGCAACCGAAACAGAGAGG-3′, reverse 5′-TTTGCAGGCCCCACATAGTT-3′), and GAPDH (forward primer: 5′-CCCCTTCATTGACCTCAACTACATGG-3′ reverse primer: 5′-GCCTGCTTCACCACCTTCTTGATGTC-3′).

### 2.10. Western Blotting for Protein Expression Determination of TLR4, NF-*κ*B, HMGB-1, and Caspase-3

Hepatic tissues were homogenized in RIPA lysis buffer, and quantitative protein analysis was determined by Bradford protein assay kit (Bio Basic Inc., Ontario, Canada). Twenty *μ*g of extracted protein from the liver of all studied groups was separated by SDS-PAGE on 4–20% polyacrylamide gradient gels and electroblotted onto polyvinylidene difluoride (PVDF) membranes using Bio-Rad Trans-Blot Turbo. After incubation in 5% nonfat dry milk, Tris-HCl, 0.1% Tween 20 for 1 hr, primary antibodies were added and incubated at 4°C overnight. The primary antibodies used were TLR4, HMGB1, NF-*κ*B, and caspase-3 (Santa Cruz Biotechnology, Santa Cruz, CA, USA). Appropriate secondary antibodies were incubated for 2 hr at room temperature. After being washed twice in 1 × TBS-T, densitometric analysis of the immunoblots was performed in all studied samples against the control sample of housekeeping protein beta-actin by protein normalization on the ChemiDoc MP imaging system.

### 2.11. Histopathological Examination and Scoring System

Small pieces of liver tissue were placed in a 10% neutral buffered formalin solution and embedded in paraffin. Tissue sections of five *μ*m thick were cut and then stained with hematoxylin and eosin (H&E) and then examined blindly by a pathologist using a light microscope. Liver sections were examined, 10 fields per section, and scored from 0 to 5 for hemorrhage, sinusoidal congestion, degeneration of hepatocyte, and parenchymal necrosis as follows: 0: when no pathological changes are found, 1: when the lesion is observed in <10% of fields, 2: when the lesion is observed in 11–25% of fields, 3: when the lesion is observed in 26–50% of fields, 4: when the lesion is observed in 51–75%, and 5: when the lesion is observed in >75% of fields [29–32]. At the end, the total scores were calculated for each rat by a pathologist.

### 2.12. Statistical Analysis

Normality of sample distribution was tested with Kolmogorov–Smirnov test. The one-way analysis of variance (ANOVA) followed by Bonferroni multiple tests was used to assess the differences between groups for measured markers. Results are expressed as mean ± SD. For histopathology, variables did not fit to the normal distribution; thus, nonparametric comparisons were performed by chi-square tests. Significance was predefined as *p* ≤ 0.05. Statistical analysis was done by using the computer software SPSS version 20 (Chicago, IL, USA).

## 3. Results

### 3.1. Effect on Liver Function Tests

To assess the liver injury caused by IR, serum levels of ALT and AST were measured as illustrated in Table 1. The serum levels of ALT and AST increased significantly in both HIR and Vilda + HIR groups when compared with the sham group (*p* < 0.05). In addition, pretreatment with Vilda in the Vilda + HIR group showed a marked decrease in serum levels of ALT and AST compared to that in the HIR group (*p* < 0.05).

### 3.2. Effect on Hepatic Oxidative and Nitrosative Stress Markers

The HIR produced a significant elevation in hepatic MDA and NO levels along with a significant reduction in hepatic catalase contents when compared with the sham group (*p* < 0.05) as presented in Table 2. Furthermore, administration of Vilda caused a significant reduction in hepatic MDA and NO contents with a significant increase in hepatic catalase levels in comparison to the HIR group (*p* < 0.05).

### 3.3. Effect on Hepatic Inflammatory Marker

In Table 2, a significant increase in hepatic TNF-*α* levels was detected in the HIR group compared to the sham group (*p* < 0.05). However, a significant reduction in hepatic TNF-*α* levels was observed in the Vilda + HIR group when compared with the HIR group (*p* < 0.05).

### 3.4. Effect on Hepatic mRNA Expressions of TLR4, HMGB1, and NF-*κ*B

**Figure 2:**
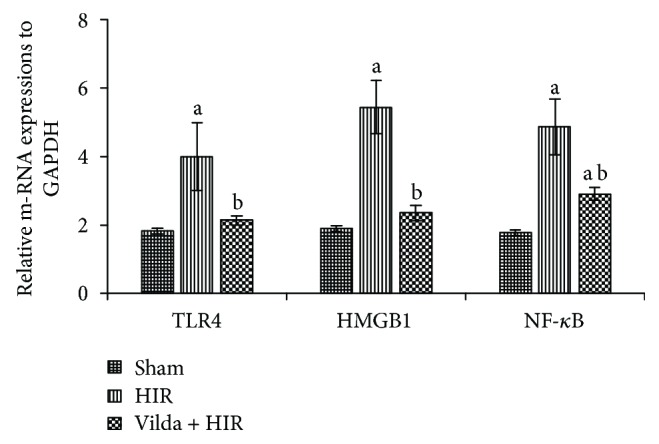
Effect of hepatic ischemia reperfusion (HIR) alone or in combination with intraperitoneal administration of vildagliptin (Vilda) (10 mg/kg for 10 days) on mRNA expressions of Toll-like receptor 4 (TLR4), high-mobility group box-1 (HMGB1), and nuclear factor-kappa B (NF-*κ*B) in the hepatic tissues of experimental rats. All data were expressed as mean ± SD. ^a^Significant difference from the sham group at *p* < 0.05. ^b^Significant difference from the HIR group at *p* < 0.05.

Figure 2 illustrated a marked upregulation of TLR4, HMGB1, and NF-*κ*B hepatic mRNA expressions by 1.2-fold, 1.9-fold, and 1.7-fold, respectively, in the HIR group when compared with the sham group (*p* < 0.05). A significant downregulation of these expressions by 46.6%, 56.9%, and 40.5%, respectively, were determined while administering Vilda in the Vilda + HIR group in comparison to the HIR group (*p* < 0.05).

### 3.5. Effect on Hepatic Protein Expressions of TLR4, HMGB1, and NF-*κ*B

**Figure 3:**
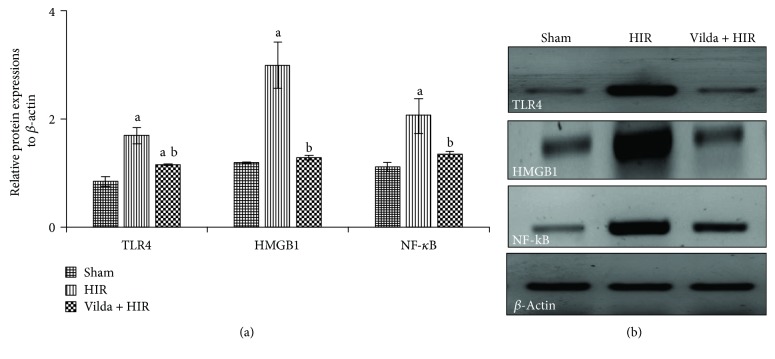
Effect of hepatic ischemia reperfusion (HIR) alone or in combination with intraperitoneal administration of vildagliptin (Vilda) (10 mg/kg for 10 days) on quantification data (a) and protein expressions of Toll-like receptor 4 (TLR4), high-mobility group box-1 (HMGB1), and nuclear factor-kappa B (NF-*κ*B) detected by Western blotting (b) in the hepatic tissues of experimental rat groups. All data were expressed as mean ± SD. ^a^Significant difference from the sham group at *p* < 0.05. ^b^Significant difference from the HIR group at *p* < 0.05.

Figure 3 explored that the HIR caused a significant upregulation of TLR4, HMGB1, and NF-*κ*B hepatic protein expressions by 1-fold, 1.5-fold, and 0.8-fold, respectively; however, a marked downregulation of these protein expressions was noticed in the Vilda + HIR group by 32%, 57.2%, and 34.6%, respectively, compared to the HIR group (*p* < 0.05).

### 3.6. Effect on Hepatic Apoptotic Marker

**Figure 4:**
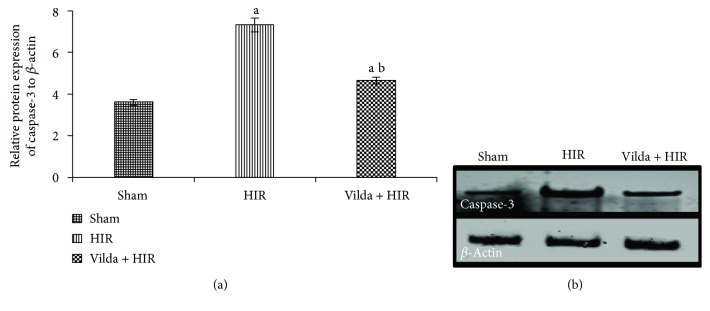
Effect of hepatic ischemia reperfusion (HIR) alone or in combination with intraperitoneal administration of vildagliptin (Vilda) (10 mg/kg for 10 days) on quantification data (a) and protein expression of caspase-3 detected by Western blotting (b) in the hepatic tissues of experimental rat groups. Data were expressed as mean ± SD. ^a^Significant difference from the sham group at *p* < 0.05. ^b^Significant difference from the HIR group at *p* < 0.05.

To evaluate the apoptosis after HIR injury, caspase-3 was determined by Western blotting assay (Figure 4). Caspase-3 protein expression was upregulated in the HIR group by 102.8% when compared to the sham group; however, it was markedly suppressed in the Vilda + HIR group by 36.5% in comparison to the HIR group (*p* < 0.05).

### 3.7. Histopathological Examination

**Figure 5:**
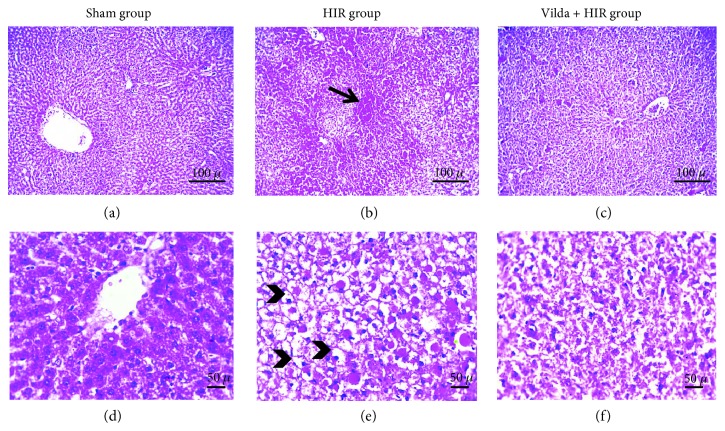
Liver sections in all groups stained with H&E with low ×100 and high ×200 magnification powers. The sham group showed normal histology (a, d). The HIR group showed congestion, focal area of hemorrhage (arrow), hydropic degeneration in hepatocytes, and necrosis with pyknotic nuclei (arrowheads) (b, e). The Vilda + HIR group showed only mild hydropic degeneration in hepatocytes (c, f).

Liver histopathological findings of the liver tissue from all groups were presented in Figure 5. The sham group showed normal pathology (Figures 5(a) and 5(d)). The HIR group histopathology was characterized by necrosis with hepatocyte nuclei which became pyknotic, small, dense, irregular, and more basophilic. Degeneration of hepatocytes was remarkable. Engorged sinusoids with blood and focal areas of hemorrhages were observed (Figures 5(b) and 5(e)). In the Vilda + HIR group, mild degeneration of hepatocytes was seen in all fields (Figures 5(c) and 5(f)). Both hepatocyte nuclei and hepatic cords maintained their normal shapes. Occasionally, sinusoidal congestion was seen.

### 3.8. Histopathological Scoring Results

According to the scoring results presented in Table 3, Pearson chi-square for sinusoidal congestion, hemorrhage, hepatocyte degeneration, and parenchymal necrosis between the sham, HIR, and Vilda + HIR groups was significant (*p* < 0.001, *p* < 0.05, *p* < 0.001, and *p* < 0.001, respectively).

## 4. Discussion

The HIR injury is considered as an important issue influencing the outcomes of liver surgeries including transplantation. It demonstrates sequential events with marked biochemical and pathological alterations that end with hepatocellular injury and damage [33, 34].

Our study exhibited that the HI for 45 minutes followed by 3 hours of reperfusion led to the liver injury which is assessed by marked elevation of liver enzymes ALT and AST levels indicating damage in the cell membrane of the liver and this is in accordance with previous studies [25, 35, 36]. Moreover, hepatic necrosis, sinusoidal congestion, hemorrhage, and hepatocyte degeneration are the characteristic pathological changes seen after HIR [24, 34, 37].

The Vilda ameliorated markedly the increase of liver enzymes as well as the histopathological changes in the liver tissue that occurred after HIR. It decreased significantly the elevated levels of the liver enzymes in the models of cyclosporine A-induced hepatotoxicity [38] and nonalcoholic fatty liver [39] confirming its hepatoprotective effect. Moreover, the protective effect of Vilda was documented before in I/R injury that occurred in the kidney [19, 40], heart [41], and lung [42]; however, our study is the first to address its protective effect against I/R injury in the liver.

It was documented that hepatic ischemia followed by reperfusion led to reactive oxygen species (ROS) production which was ultimately involved in the pathogenesis of HIR by either attacking various biomolecules or activating the innate system [43, 44]. Thus, to assess the oxidative damage MDA, the lipid peroxidation product was measured in addition to the antioxidant enzyme catalase that acts as a free radicle scavenger counteracting the damaging effect of ROS [44–46]. Our study documented a significant increase in MDA level alongside with a marked decline in catalase level in hepatic tissue of rats which underwent HIR injury when compared with the sham group, and this result coincided with other studies [44, 47].

Pretreatment with Vilda showed a significant reduction in hepatic MDA content reflecting a decline in the oxidative stress state. In addition, Vilda showed a marked elevation in catalase hepatic content indicating its antioxidant effect. It was reported before that the DPP-4 inhibitor Vilda attenuates the ROS production and its subsequent oxidative damage in various animal models by declining the lipid peroxidation marker level along with rising the levels of antioxidant enzymes [38, 39, 48, 49].

In addition to oxidative stress, the pivotal role of nitrosative stress and inflammation in the pathogenesis of HIR was emphasized [50]. A significant increase in hepatic NO and TNF-*α* levels was observed in the HIR group compared to sham rats, and this finding was inconsistent with other studies [36, 50]. The NO has a dual effect on the liver physiology during ischemic injury depending on the isoform of NO synthase (NOS). The NO overproduction induced by iNOS could have a damaging effect to hepatic tissue due to its reaction with superoxide anion which led to the formation of peroxynitrite radical with the oxidative toxic effect that added further injury to the liver [24, 51]. Moreover, it has been documented that during the reperfusion phase, the activated Kupffer cells released proinflammatory cytokines including TNF-*α* which is regulated by NF-*κ*B [36] and led to the upregulation of iNOS expression [52].

Administration of Vilda showed a marked decline in hepatic NO and TNF-*α* contents compared to the nontreated HIR group. The anti-inflammatory activity of Vilda through reduction of TNF-*α* serum levels and mRNA expression as well as NO levels was confirmed in various models [38, 52–55]. The antioxidant and anti-inflammatory effects of the DPP-4 inhibitor Vilda may be attributed to the increased levels of GLP-1 that led to the GLP-1 receptor activation [53] that could protect against the mitochondrial dysfunction [55].

The involvement of the TLR4/NF-*κ*B signaling pathway in the pathogenesis of hepatic I/R injury was reported in several studies [13, 36, 56, 57]. Our results revealed that the mRNA and protein expressions of hepatic TLR4 and NF-*κ*B were markedly elevated in the group of HIR and these results coincided with other studies [13, 56, 57]. The TLR4 signaling leads to the activation of the nuclear transcription factor NF-*κ*B which initiates the release of various numbers of proinflammatory mediators including TNF-*α* [58, 59]. The upregulation of TLR4 was associated with the rise in TNF-*α* causing further liver damage and injury [36, 59]. That is why the inhibition of the activity of the TLR4/NF-*κ*B signaling pathway by drugs could afford beneficial effects for the liver and presenting a potential therapy against HIR [13, 57].

There is no previous study that showed the effect of Vilda on the hepatic TLR4/NF-*κ*B signaling pathway during I/R injury; however, it was found that sitagliptin which is one member of the DPP-4 inhibitor family significantly reduced the renal TNF-*α* and NF-*κ*B after renal I/R injury [16, 60]. Furthermore, Vilda reduced significantly the hepatic NF-*κ*B in a model of cyclosporine A-induced hepatotoxicity [38]. Lee and his coauthors confirmed the anti-inflammatory effect of Vilda via reduction of TLR-4 expression [61]. The effect of Vilda was through DPP-4 inhibition and GLP-1 elevation that led to the suppression of the TLR-4/NF-*κ*B signaling pathway [61, 62].

Our results showed marked elevation in mRNA and protein expressions of hepatic HMGB1 in the HIR group compared to the sham group. Several studies are in agreement with our results [57, 63, 64]. HMGB1 is a nuclear protein which plays a crucial role in mediating hepatic inflammation, injury, and necrosis. Thus, it is considered as a marker of cellular damage reflecting cellular structural integrity during I/R [63].

The activation of Kupffer cells and neutrophils that occurred during HIR led to the release of ROS and proinflammatory cytokines like TNF*α* and HMGB1 which are released from either necrotic liver cells or activated Kupffer cells and neutrophils [63, 65]. Moreover, a positive correlation between HMGB1 expression and proinflammatory cytokine levels was reported. In addition, it was found that TLR4 enhanced the HMGB1 expression through NF-*κ*B pathway activation [64].

Blocking the release of HMGB1 has been shown to protect hepatocellular damage during I/R in animal models indicating its therapeutic target in HIR [63]. The Vilda treatment showed a significant reduction of mRNA and protein expressions of HMGB1 when compared with the HIR group confirming its ability in ameliorating the hepatic injury.

To investigate the role of apoptosis in liver injury induced by HIR, the caspase-3 protein expression was determined in the hepatic tissue. Our study showed that HIR was associated with a significant upregulation in hepatic caspase-3 protein expression compared to the sham group and this was in agreement with others [36, 66]. The ischemic liver is characterized by cellular apoptosis and programmed cell death, which is recognized by the caspase family in which caspase-3 is the executive caspase [67]. Growing evidence suggested that TNF-*α* and oxidative stress played a crucial role in magnifying the apoptosis in the ischemic liver [23, 36].

Treatment with Vilda exhibited a marked downregulation of hepatic caspase-3 expression when compared with HIR rats. A recent study documented that Vilda was able to decrease caspase-3 in a model of cardiac I/R injury [41].

## 5. Conclusions

Vildagliptin ameliorated for the first time the hepatic damage induced by HIR evident by (a) improvement in the liver function tests, (b) suppression of oxidative stress via reduction of elevated hepatic levels of MDA and NO along with increment of the reduced catalase hepatic level, (c) blocking of the hepatic inflammatory cytokine TNF-*α*, (d) downregulation of gene and protein expressions of TLR4, HMGB1, and NF-*κ*B, and (e) reduction of apoptosis by downregulation of hepatic protein expression of the apoptotic marker caspase-3. This study suggests that the use of Vilda could be beneficial in liver surgeries associated with I/R; however, further studies are encouraged to validate our findings.

## Figures and Tables

**Table 1:** Effect of hepatic ischemia/reperfusion (HIR) alone or in combination with intraperitoneal administration of vildagliptin (Vilda) (10 mg/kg for 10 days) on serum alanine transaminase (ALT) and aspartate transaminase (AST) levels in experimental rats. All data were expressed as mean ± SD.

Group	ALT (U/L)	AST (U/L)
Sham	23.52 ± 1.12	47.26 ± 4.6
HIR	86.06 ± 4.71^a^	116.6 ± 8.7^a^
Vilda + HIR	37 ± 5.24^ab^	65 ± 4.3^ab^

^a^Significant difference from the sham group at *p* < 0.05. ^b^Significant difference from the HIR group at *p* < 0.05.

**Table 2:** Effect of hepatic ischemia/reperfusion (HIR) alone or in combination with intraperitoneal administration of vildagliptin (Vilda) (10 mg/kg for 10 days) on oxidative stress malondialdehyde (MDA) and catalase, nitrosative stress nitric oxide (NO), and inflammatory mediator tumor necrosis factor alpha (TNF-*α*) levels in the liver of experimental rats. All data were expressed as mean ± SD.

Group	MDA (nmol/mg protein)	Catalase (nmol/min/g tissue)	NO (nmol/mg protein)	TNF-*α* (pg/mg protein)
Sham	7 ± 0.75	43.1 ± 1.56	2.12 ± 0.52	235 ± 11.57
HIR	24.22 ± 1.96^a^	18.08 ± 1.5^a^	7.8 ± 0.57^a^	818.2 ± 75.44^a^
Vilda + HIR	15.56 ± 0.8^b^	29.8 ± 3.29^ab^	5.02 ± 0.37^ab^	431.1 ± 63.2^ab^

^a^Significant difference from the sham group at *p* < 0.05. ^b^Significant difference from the HIR group at *p* < 0.05.

**Table 3:** Comparison of histopathological scores between Sham, HIR, and Vilda + HIR groups *n* (%).

Parameters	Sham	HIR	Vilda + HIR	*χ* ^2^	*p*
Sinusoidal congestion					
Score 0	10 (100%)	0 (0%)	6 (60%)	37.5	<0.001
Score 1	0 (0%)	0 (0%)	4 (40%)		
Score 3	0 (0%)	2 (20.0%)	0 (0%)		
Score 4	0 (0%)	4 (40%)	0 (0%)		
Score 5	0 (0%)	4 (40%)	0 (0%)		
Hemorrhage					
Score 0	10 (100%)	2 (20%)	10 (100%)	21.8	<0.05
Score 1	0 (0%)	4 (40%)	0 (0%)		
Score 2	0 (0%)	2 (20%)	0 (0%)		
Score 3	0 (0%)	2 (20%)	0 (0%)		
Hepatocyte degeneration					
Score 0	10 (100%)	0 (0%)	0 (0%)	43.0	<0.001
Score 1	0 (0%)	0 (0%)	2 (20%)		
Score 2	0 (0%)	0 (0%)	2 (20%)		
Score 3	0 (0%)	0 (0%)	2 (20%)		
Score 4	0 (0%)	4 (40%)	2 (20%)		
Score 5	0 (0%)	6 (60%)	2 (20%)		
Parenchymal necrosis					
Score 0	10 (100%)	0 (0%)	10 (100%)	30	<0.001
Score 4	0 (0%)	4 (40%)	0 (0%)		
Score 5	0 (0%)	6 (60%)	0 (0%)		

## Data Availability

Data used to support the findings of this study are available upon request.
